# Research on Defects Inspection of Solder Balls Based on Eddy Current Pulsed Thermography

**DOI:** 10.3390/s151025882

**Published:** 2015-10-13

**Authors:** Xiuyun Zhou, Jinlong Zhou, Guiyun Tian, Yizhe Wang

**Affiliations:** 1 School of Automation Engineering, University of Electronic Science and Technology of China, Chengdu 611731, China; E-Mails: zhouxy@uestc.edu.cn (X.Z.); g.y.tian@newcastle.ac.uk (G.T.); wangyizhe617525@163.com (Y.W.); 2 School of Electrical and Electronic Engineering, Newcastle University, Newcastle upon Tyne NE1 7R, UK

**Keywords:** flip-chip solder balls, eddy current pulsed, infrared thermography, defect detection

## Abstract

In order to solve tiny defect detection for solder balls in high-density flip-chip, this paper proposed feasibility study on the effect of detectability as well as classification based on eddy current pulsed thermography (ECPT). Specifically, numerical analysis of 3D finite element inductive heat model is generated to investigate disturbance on the temperature field for different kind of defects such as cracks, voids, *etc.* The temperature variation between defective and non-defective solder balls is monitored for defects identification and classification. Finally, experimental study is carried on the diameter 1mm tiny solder balls by using ECPT and verify the efficacy of the technique.

## 1. Introduction

In many forms of advanced packaging, solder bump technology has become a reliable electrical interconnection method. The solder bump technology provides decreased package size, greater I/O density and larger speed of signal propagation [1]. However, due to the coupling active infection of thermal, electrical, fluid, motion and other multi-physics, solder joints is suffered such as cracks, voids and other defects, leading to false alarm. Solder joint defect detection and reliability assessment has become one of the key issues to be solved in IC manufacturing technology [2].

Defects detection methods of solder joints include contact and non-contact techniques [3]. Contact detection methods can well detect short circuit and open circuit defects, but unable to realize the identification and location of defects. Non-contact methods consist of automated optical inspection [4], X-ray detection [5] and scanning acoustic microscope (SAM) detection method [6]. Automated optical inspection mainly detects coplanar defect. However, it cannot detect hidden defect of solder joint X-ray detection can distinguish the inherent characteristics of the solder joints whereas it cannot distinguish vertical overlapping features, and the equipment is expensive. SAM can qualitatively analyse the solder joint defect. However, it causes misjudgement due to the difference of package structure.

Infrared camera non-destructive testing technology has been widely used in the field of electronic measurement and inspection. Chai *et al.* put forward active transient thermography for detecting flip chip solder balls [7]. The flip chip is coupled with the electrode pairs and injected working current. When defects exist in solder ball, the resistance of defective solder ball is significantly higher than that of the normal solder ball, resulting in temperature anomalies. Therefore, according to the light and dark areas of thermal image of IR imaging senor, the existence and location of defects can be detected. Whereas, this method is only available for void defects and partial cracks detection.

In this paper, a novel approach based on eddy current pulsed thermography was investigated for defect inspection of solder balls. Both simulation and experiment on the defects of solder joint are carried out. Eddy current pulsed thermography is an emerging detection method, which combines the advantages of eddy current testing and thermal imaging technology. Through numerical and experimental studies, it can be found that ECPT has both high spatial resolution and sensitivity when assessing both electrical and thermal properties [8,9,10,11,12,13,14,15]. Currently, this technique has been applied to conductive composite defect inspection and classification [16,17], crack detection of rolling contact fatigue of rail tracks [18], glass fibre reinforced polymer specimen detection [19] and power electronic devices [20]. However, the size of test specimen of these research is relatively large in these references, thus employing ECPT technology to study the detectability on small defects and features of tiny structures (such as solder ball) is the main target of this article. The rest of the paper is organized as follows. Firstly, the detection mechanism is analysed in Section 2. Then, simulation researches and analysis are introduced in Section 3, which is followed by experimental studies and discussion in Section 4. Finally, conclusions and future work are outlined in Section 5.

## 2. Detection Principle

**Figure 1:**
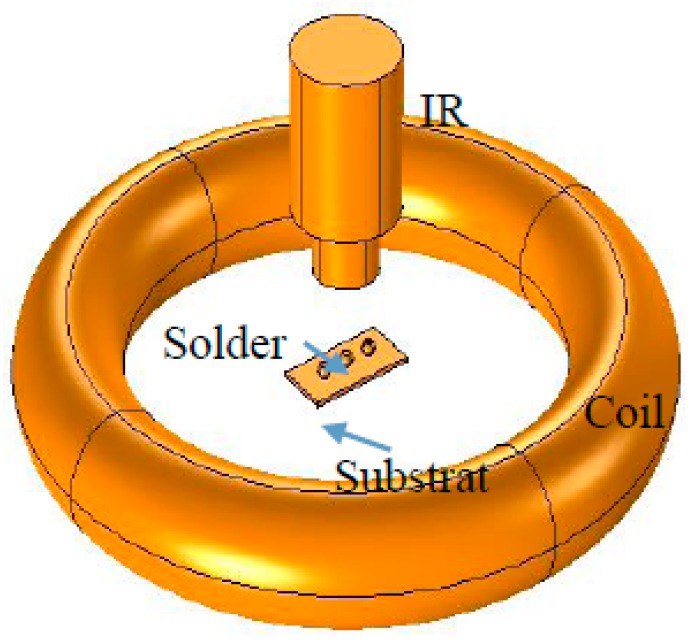
The schematic diagram of pulsed eddy current thermography.

Figure 1 is the schematic diagram of ECPT. According to Faraday’s law of electromagnetic induction, when an alternating current is applied to a coil, an alternating magnetic field appears around the coil. The tested solder balls located in the alternating magnetic field induce eddy current. The law of electromagnetic induction is described by Equations (1) and (2):

(1) $$(jw\sigma -w^{2}\varepsilon )A+\nabla \times (\mu^{-1}\nabla \times A)=J_{e}$$

(2) B=∇×A

where *B* is the magnetic flux density, *A* is the magnetic vector potential, *J*_e_ is the current density, *w* is the angular frequency, *σ* is the electrical conductivity, µ is permeability and *ε* is permittivity. According to Joule’s law, when there is current in the conductor, the conductor generates Joule heat (or resistive heating). The sum of the generated Joule heat *Q* is proportional to the square of the magnitude of the electric current density *J*_e_ .Current density, in turn, is proportional to the electric field intensity vector *E*. The relationship between *Q*, *J*_e_ and *E* is governed by following Equation (3):

(3) $$Q=\frac{1}{\sigma }|J_{e}{|}^{2}=\frac{1}{\sigma }|\sigma E{|}^{2}$$

At the same time, Joule heat spreads in the solder ball inside, the propagation law follows Equation (4):

(4) $$\rho C_{p}\frac{\partial T}{\partial t}-\nabla (k\nabla T)=Q$$

where *ρ* is the density of the material is, *C*_p_ is the heat capacity, *k* is the thermal conductivity.

Figure 1 shows the diagram of ECPT. The excitation signal generated by the excitation module is a small period of high frequency current. The current in the coil will induce the eddy currents and generate the resistive heat in the conductive material. The heat will diffuse in time until the heat reaches equilibrium in the material. If a defect (e.g., crack, fatigue region) is present in the conductive material, eddy current distribution as well as heat diffusion process will vary. Consequently, the spatial distribution of temperature on the surface of material and the temperature transient response will show the variation, which is captured by an infrared camera.

## 3. Simulation Model and Numerical Studies

Numerical simulation analysis is an effective tool for the study of multi-physics for ECPT on conductive sample [21]. This paper takes Comsol Multiphysics as the 3D simulation platform. The study will concentrate on the common defects (void or crack) in solder ball of flip chip. Sn60%Pb40% solder balls with a diameter of 0.4 mm were used in this simulation study, and the interval between solder balls was 0.6 mm. The void radius and crack size were set to 90 µm, 200 µm × 100µm × 60µm respectively. A cuboid with the size of 2 mm × 1 mm × 0.2 mm replaces the substrate to simplify the model. The coil was a copper inductor with 30 mm outer diameter and 18 mm inner diameter one-turn. The material proprieties employed in the simulation are listed in Table 1.

**Table 1:** Properties of the materials

Materials	Thermal Conductivity	Density	Specific Heat Capacity	Electrical Conductivity	Relative Permeability
	K (W/(m∙k) )	Ρ (kg/m^3^)	Cp (J/(kg∙k))	Σ (S/m)	μ
Solder	50	9000	150	6.67 × 10^6^	1
Air	0.023	1.29	1000	0	1
Substrate	0.3	1900	1369	0.004	1

Since the size of solder ball and defect is tiny, high temperature overlarge generated by excitation current can melt the solder ball. On the other hand, solder cannot produce enough heat because of too small an excitation current; hence, the detectability performance of the IR camera will be influenced. Meanwhile, the current frequency has also an effect on skin depth on the upper surface of the solder balls. What’s more, the relative position of the coil and the solder balls determines the direction of magnetic field and the effects of defect detection. Thus, it is necessary to study the impact factors of detectability which include excitation current density, excitation frequency and coil position. The following section investigates the impact factors for testing study.

### 3.1. Simulation Research of Different Defects

**Figure 2:**
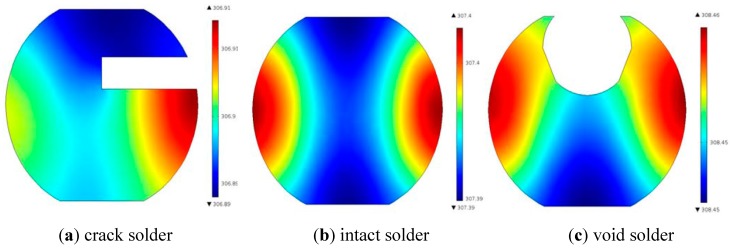
Profile temperature distribution.

The simulation model was established with the coil being placed horizontally. The current density and frequency were set to 1.26 × 10^−7^ A/m^2^, 256 kHz individually. Due to the presence of void and crack, heat generation and conduction are both different, leading to the temperature difference of solder balls in surface and internal region. The profile temperature distribution of the crack solder ball, the intact solder ball and the void solder ball is shown in Figure 2. It can be seen that the internal temperature distributions of three types solder ball are obviously different.

**Figure 3:**
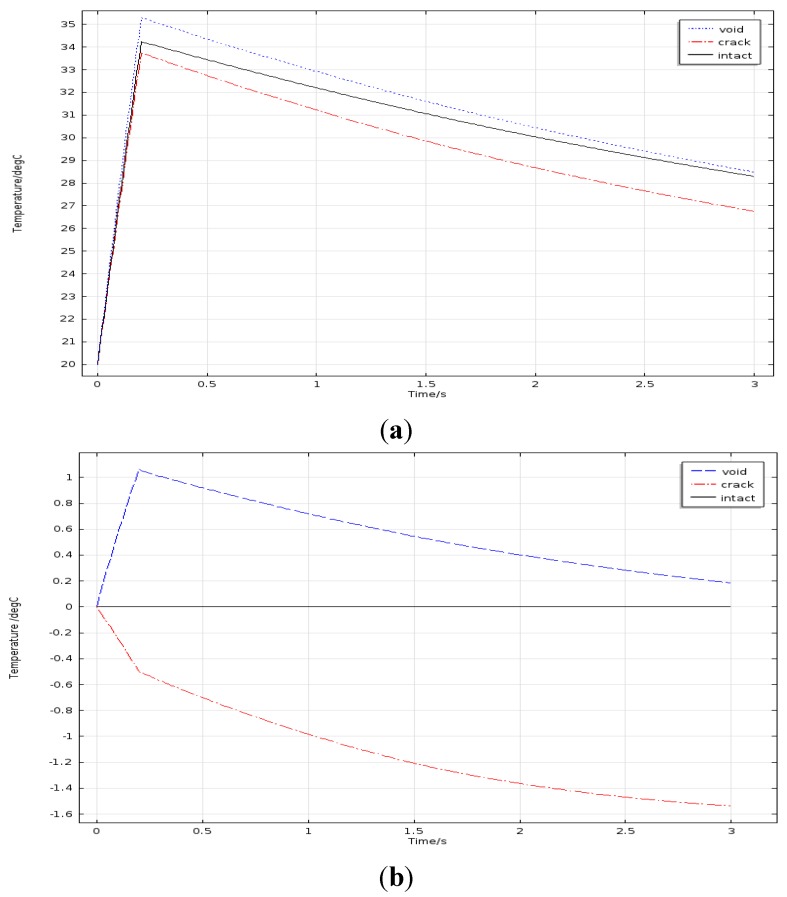
(**a**) Temperature curves of solder balls; (**b**) Temperature difference curves.

Average temperature of three types solder ball was extracted from the upper surface and the time-temperature curves are shown in Figure 3a. It can be seen from Figure 3a that the temperature increases linearly in 0–0.2 s and reaches maximum point at 0.2 s, and then declines slowly. Compared the defective solder balls with the intact solder ball, the temperature variation trends are the same.

In order to make the differences between the temperature histories of the defective solder balls (T_def_) and the intact one (T_ref_) more evident, T_def_ − T_ref_ is considered. Figure 3b shows the temperature differences (T_def_ − T_ref_ ) along with the time. It can be seen from Figure 3b, the temperature difference between the void solder ball and the intact solder ball is positive, indicating the upper surface temperature of the void solder ball is higher than that of the intact solder ball; the temperature difference between the crack solder ball and the intact solder ball is negative, indicating that the upper surface temperature of the crack solder ball is lower than the intact solder ball.

**Figure 4:**
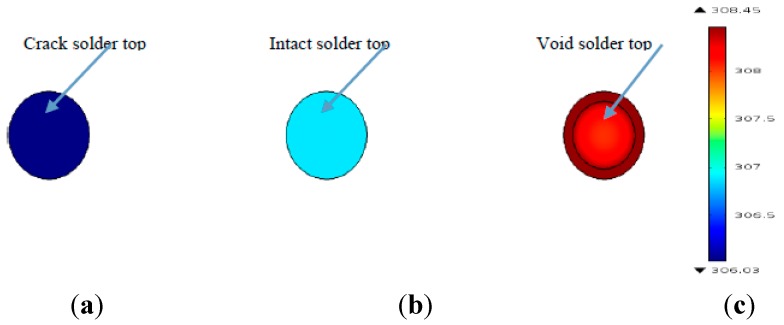
The upper surface temperature images of three solder ball types: (**a**) Crack solder top; (**b**) Intact solder top; (**c**) Void solder top.

Figure 4 shows the temperature distribution of the upper surface of three types solder ball at 0.2 s. It can be seen from Figure 4, that the upper surface temperature of the crack solder ball is the lowest in three solder ball types, the dark spot is formed in the corresponding top region. The upper surface temperature of the void solder ball is the highest. In addition, it forms an annular image with the lower temperature middle region. For the crack solder ball, the temperature forms as light colour spot. Thus from Figure 3 and Figure 4, different defects in solder ball can be distinguished according to temperature curves and temperature images.

**Figure 5:**
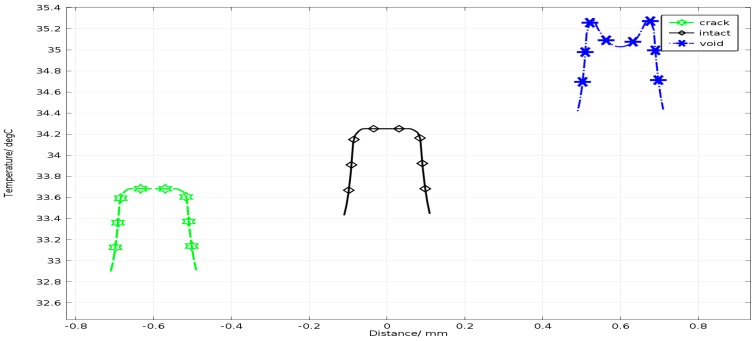
Temperature distribution at each point along the straight.

In order to illustrate the temperature distribution map explicitly, the temperature straight lines with a span of 174 µm were made at the upper surface of each solder ball, as shown in Figure 5. This shows that the upper surface temperature of the crack solder ball is the lowest among three types solder balls, presenting the dark spot in Figure 4a. The upper surface temperature of the void solder ball is the highest, but the top edge of the void solder ball is a high-temperature region in contrast with the low-temperature region in the void middle region, forming a trough in the void location, which is inconsistent with conclusion described in the literature [2]. Hence, it is necessary to analyse the internal temperature field of the solder ball based on pulsed eddy current excitation.

For a homogeneous field excitation, the skin depth of a magnetic field in a material is governed by Equation (5):

(5) $$\delta =\frac{1}{\sqrt{\pi f\mu \sigma }}$$

Where *δ* is the electric conductivity, *µ* is the magnetic conductivity, and *f* is the frequency of the pulsed excitation. The calculated results corresponding to a magnetic frequency of 256 kHz reveal that the skin depth of the solder ball is around 385 µm which is larger than the radius of the simulation solder ball. Therefore, the temperature difference of the solder ball is mainly ascribed to the defect perturbations on eddy current field [22].

**Figure 6:**
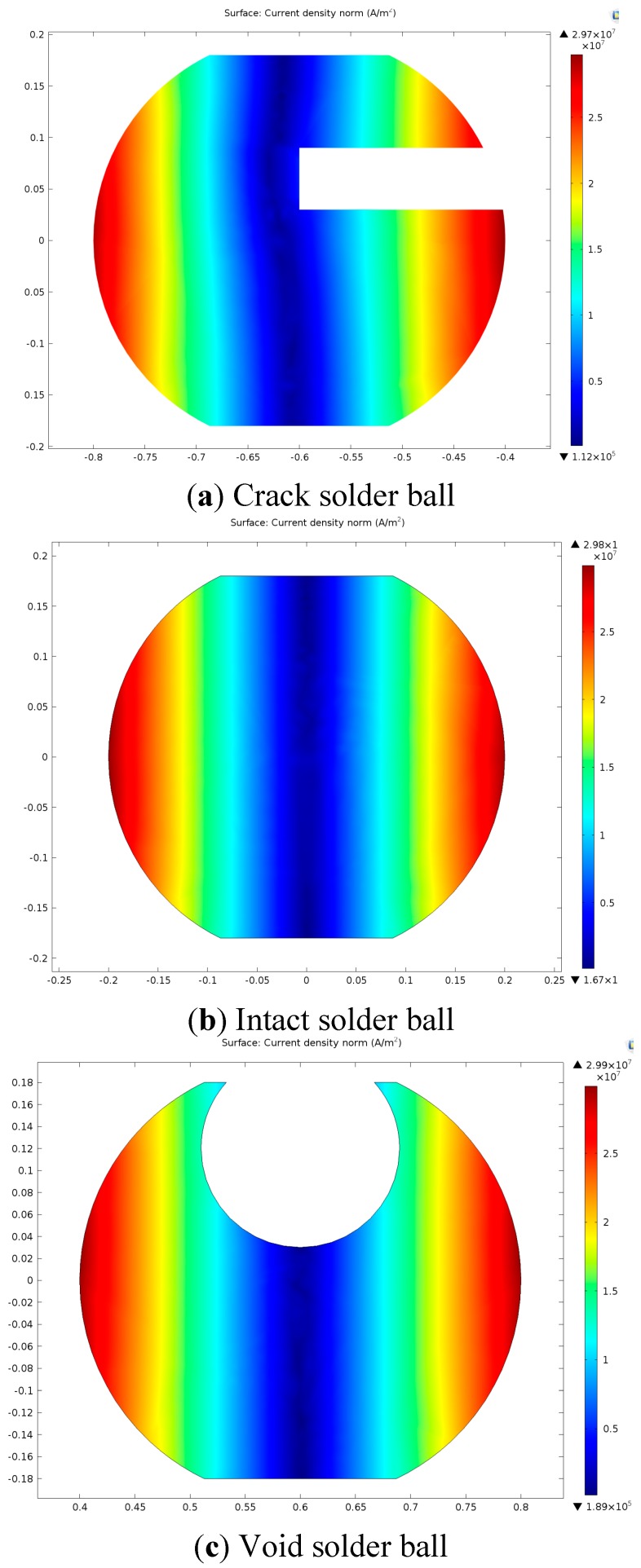
The eddy current density distribution.

The eddy current density distribution of three solder balls at 0.2 s is shown in Figure 6. The eddy current distribution regularities of the three solder balls are substantially consistent. The eddy current density gradually decreases from the surface to the centre, but the eddy current density is various in different defect positions.

According to Equation (3), the sum of induction heat is determined by the eddy current density and the electrical conductivity. Hence the heat generated on the surface is much larger than inner heat. The temperature of the solder ball can be deduced by Equation (6) [23]:

(6) $$Q=\sum C(T)\cdot \rho V\cdot (T-T_{0})$$

where *ρ* is the density of the solder ball, *V* is the volume of the solder, *C*(*T*) is the thermal capacitance, *T*_0_ is the room temperature and *T* is the temperature of the solder ball. Since the void is located in the interior of the solder ball where the eddy current density is rather minimal, it has a negligible effect on the total heat Q. However, the void reduces the volume of the solder ball, resulting in the high temperature according to the Equation (6). On the other hand, according to the theory of thermal resistance, lateral heat conduction is better than the longitudinal heat conduction when heat propagates in the solder ball [24]. Due to that the lateral heat conduction is blocked in the void part, heat aggregates on the top of the solder ball from the high-temperature region to the low-temperature regions by the vertical heat transmission, which makes the upper surface temperature of the void solder ball higher than that of the intact solder ball. For the crack solder ball, the crack exists in the near-surface region is shown in Figure 6a. In addition, the thermal resistance in the crack is rather large [25], which has a negative effect on the heat transmission from the high-temperature zone to the low-temperature zone, further leading to the lower temperature than the intact solder ball. This is also consistent with the result presented by Figure 4a.

### 3.2. The Detectability Impact Factor of Current Density and Frequency

In order to study the detectability by using reasonable current density and frequency range , a series of studies were conducted .The size of solder ball is small and the melting point of materials is 183 ºC. When the excitation current density and the frequency are too low, the inductive heat is scant and the temperature does not change obviously. The overlarge excitation current density and frequency can melt the solder balls, which can lead to failing the test. These disadvantages will influence us to analyse the results.

**Figure 7:**
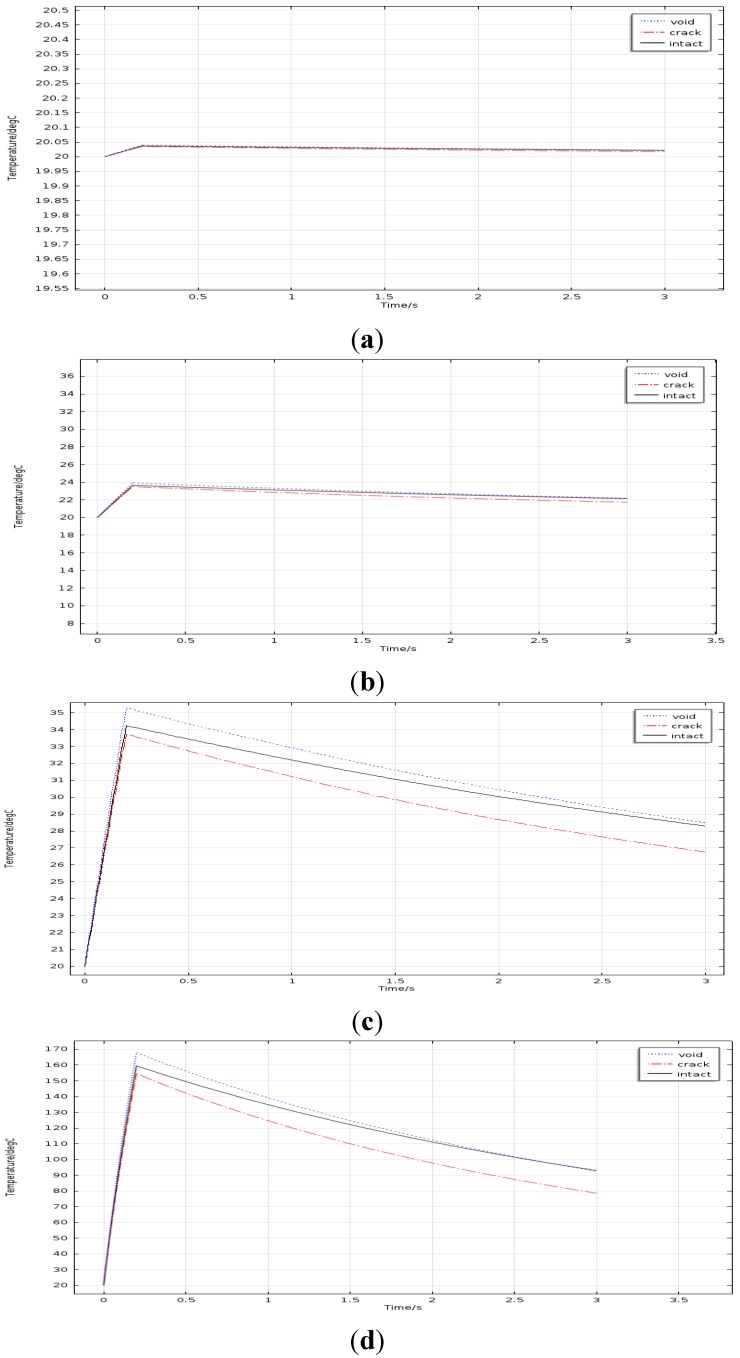
Temperature curves for different current density for (**a**) 6.3 × 10^5^ A/m^2^; (**b**) 6.3 × 10^6^ A/m^2^; (**c**) 1.26 × 10^7^ A/m^2^; (**d**) 4.41 × 10^7^ A/m^2^.

When the frequency was set to 256 kHz, simulation models were established with different excitation current density values: 6.3 × 10^5^ A/m^2^, 6.3 × 10^6^ A/m^2^, 1.26 × 10^7^ A/m^2^ and 4.41 × 10^7^ A/m^2^ respectively. The maximum current density of the experimental device is 4.41 × 10^7^ A/m^2^ and the others are obtained by reducing the current density to two-sevenths, one seventh and one seventieth of the maximum current density. Average temperature values on the top of different solder balls were extracted from each model and the time-temperature curves were depicted in Figure 7.

From Figure 7a,b, when the current density was lower than 6.3 × 10^6^ A/m^2^, the three curves are almost overlapping. It can conclude that the temperature differences between different solder balls are weeny, which makes the results be disturbed easily by environmental factors. Therefore, the current density value of less than 6.3 × 10^6^ A/m^2^ is infeasible. Figure 7d shows that when the current density is set to 4.41× 10^7^ A/m^2^, the temperature rise is closed to the melting point of solder ball, so it is not an appropriate choice. However, from Figure 7c, not only the temperature differences between solder balls are evident, but also the temperature rise is not so high. In conclusion, the proper current density should be near to 1.26 × 10^7^ A/m^2^, but it cannot exceed 4.41 × 10^7^ A/ m^2^.

**Figure 8:**
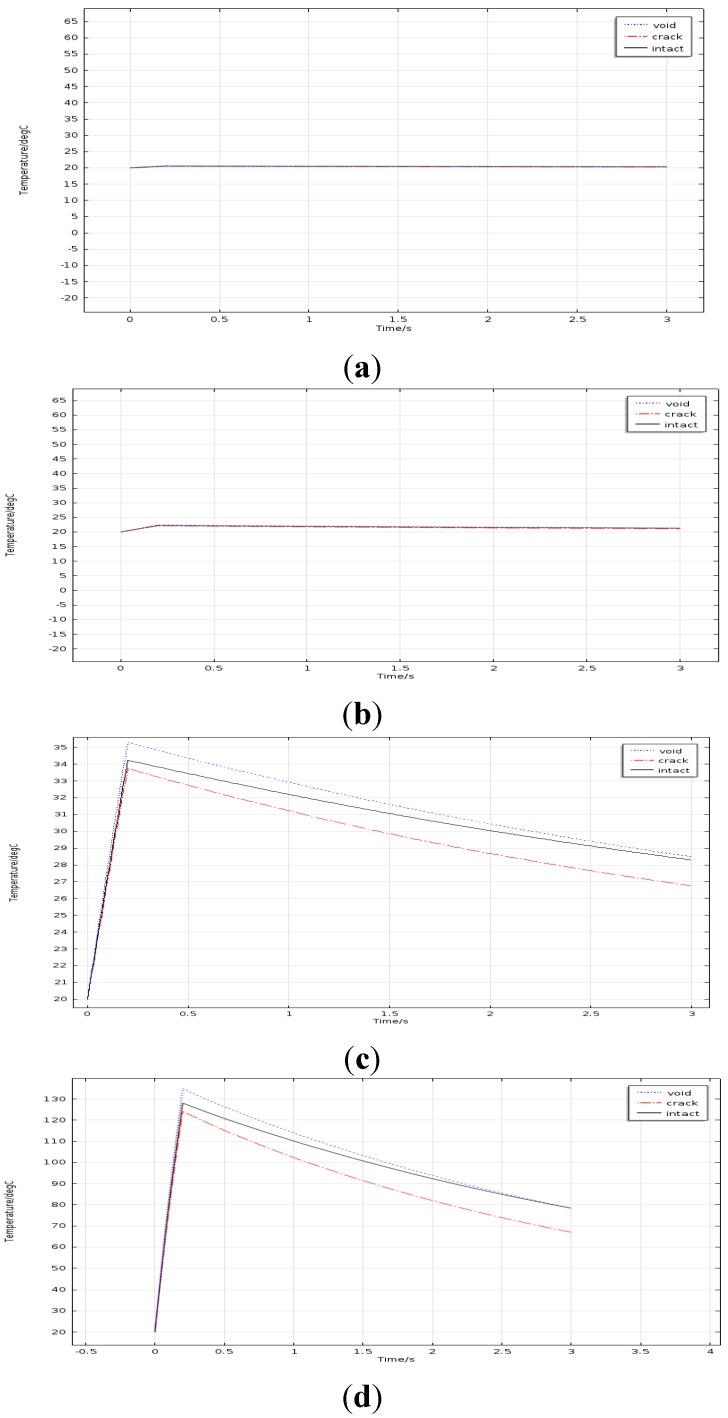
Temperature curves for different frequency: (**a**) 50 kHz; (**b**) 100 kHz; (**c**) 256 kHz; (**d**) 768 kHz.

When the current density is set to 1.26 × 10^7^ A/m^2^, simulation models were established with different frequency and time-temperature curves are depicted as shown in Figure 8. In terms of frequency, the heating current frequency is 256 KHz, which is the same as that in the previous study for process development .In order to study the reasonable frequency range, 768 kHz is chosen as the upper bound, which is 3 times as large as 256 kHz. And 100 kHz and 50 kHz which are half of 256 kHz and a quarter of 256 kHz respectively are chosen as the lower bounds. By comparing four figures in Figure 8, it can be seen the temperature rising and temperature difference of the solder ball are both evident in Figure 8c.The results of Figure 8a,b are unsatisfactory. The temperature rising in Figure 8d is too high. In conclusion, the proper frequency should be near to 256 kHz.

### 3.3. Simulations of the Relative Positions of the Coil and the Solder Balls

**Figure 9:**
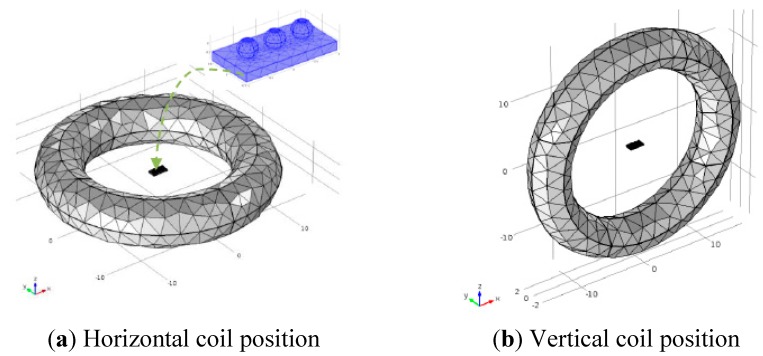
Different relative positions.

**Figure 10:**
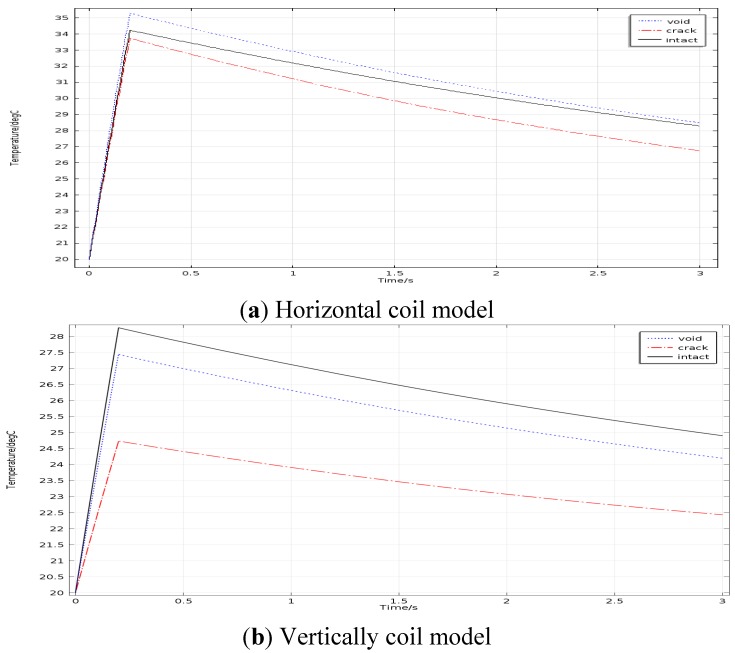
Temperature curves for each simulation model.

Two kinds of simulation model are discussed to ascertain the relative positions of the coil and the solder balls for the subsequent experiment study. The coil is placed horizontally in one model, it is placed vertically in the other one, as shown in Figure 9. The current density and frequency is set to 1.26 × 10^7^ A/m^2^ and 256 kHz .The duration of heating and heat diffusion is set to 0.2 s and 2.8 s (3–0.2 s) respectively. Average temperature values on the top of different solder balls were extracted from each model and the time-temperature curves were depicted as shown in Figure 10.

From Figure 10a, the upper surface temperature of the void solder ball is higher than that of the intact solder ball and the upper surface temperature of the crack solder ball is the lowest. Thus the three types solder ball can be distinguished intuitively in terms of upper temperature. However, the upper surface temperature of both void solder ball and the crack solder ball is lower than that of the intact solder ball as shown in Figure 10b, the crack and the hole cannot be distinguished from each other by using the temperature curves. Above all, the coil placed horizontally should be the appropriate selection.

## 4. Experimental Verification and Evaluation

**Figure 11:**
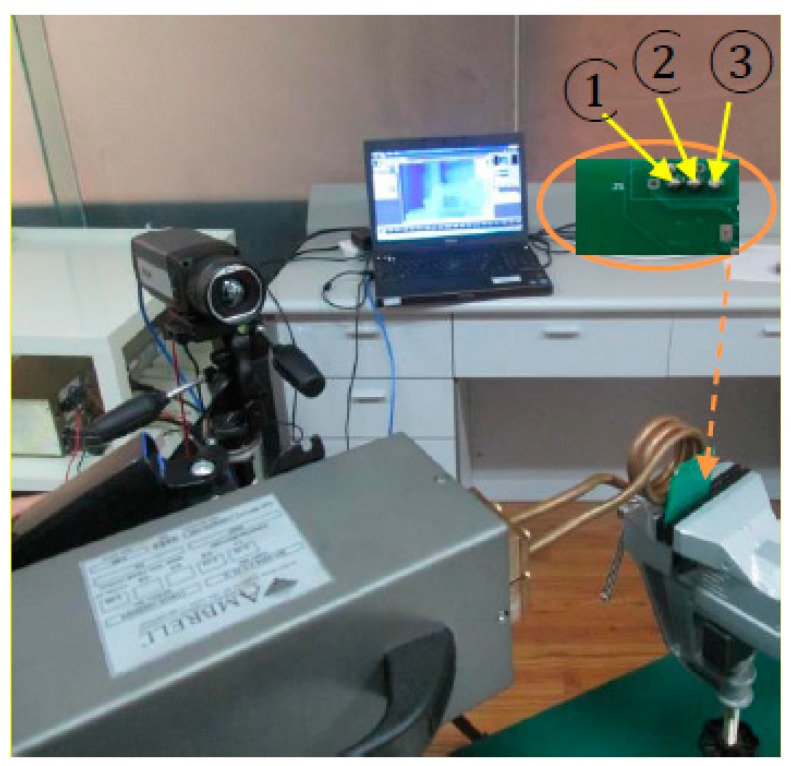
Experiment set-up, where (1), (2) and (3) represent the crack solder ball, the intact solder ball and the void solder ball respectively.

The experimental set-up is shown in Figure 11. An Easyheat 224 from Cheltenham Induction Heating (Gloucestershire, UK) is used for coil excitation. The Easyheat has a maximum excitation power of 2.4 kW, a maximum current of 400 Arms and an excitation frequency range of 150–400 kHz (380 Arms and 256 kHz are used in this study). A circular coil is constructed to apply directional excitation. This coil is made of high conductivity hollow copper tube. Water cooling of the coil is implemented to counteract direct heating of the coil. This IR camera is a Stirling cooled camera with a 320 × 256 array of 1.5–5 μm InSb detectors shown in Figure 11. The maximum 200 Hz frame rate provides 1 frame every 2.6 ms, with the option to increase the frame rate with windowing of the image.

The experiment sample was collected from Industry Company who produces VLSI (Very Large Scale Integration) with three typical types of defect. The labels (1), (2) and (3) in Figure 11 represent the crack solder ball, the intact solder ball and the void solder ball respectively. The diameter of the solder balls, which were made of Sn60%Pb40% wire, is 1mm and the interval between solder balls is 1.5 mm [26,27]. In this study, the trigger delay and heating time incentives both are 0.2 s and 3 s videos are recorded.

**Figure 12:**
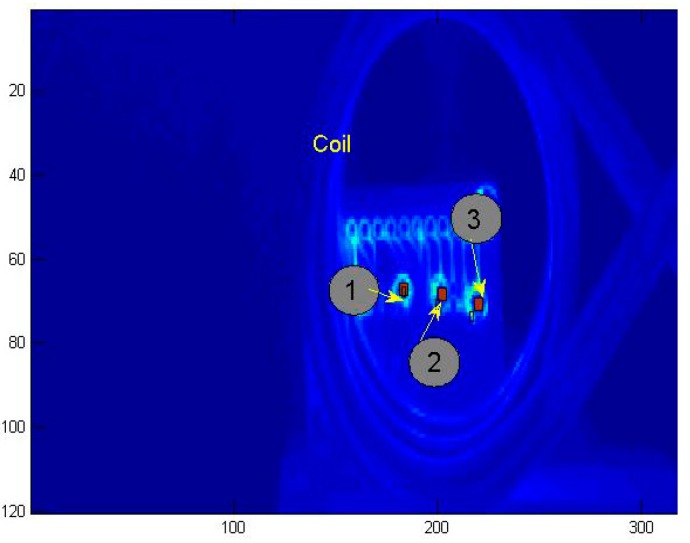
Temperature image at 0.4 s, where (1), (2) and (3) represent the crack solder ball, the intact solder ball and the void solder ball respectively.

**Figure 13:**
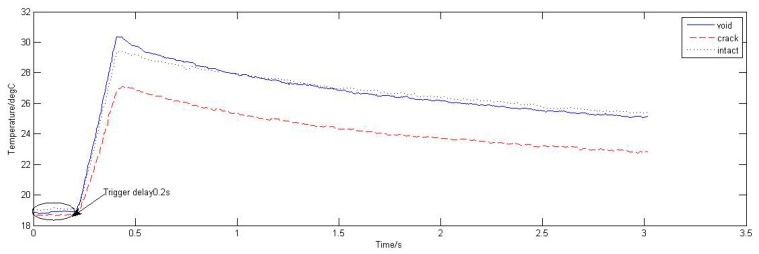
Average temperature curves.

**Figure 14:**
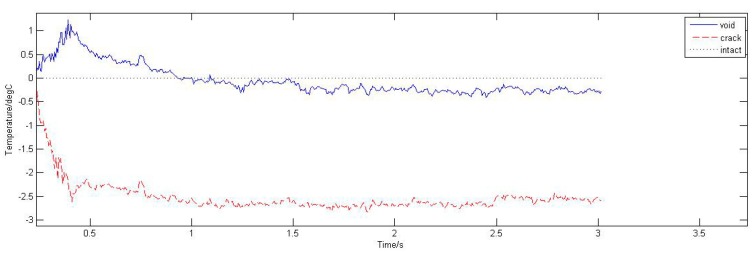
Temperature difference curves.

The temperature image of the solder balls on the PCB board at 0.4 s is depicted in Figure 12. The average temperature of each solder ball (red rectangle region pointed by arrow) was extracted at each time, and the temperature curves and the temperature difference curves were showed in Figure 13 and Figure 14 independently.

**Figure 15:**
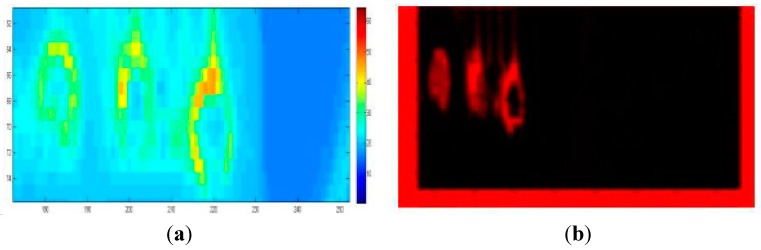
Thermal image on the solder top: (**a**) The original thermal image on the top; (**b**) The thermal image through Gauss low-pass.

In contrast with the simulation curves in the Figure 2c and Figure 7, the experimental results and the simulation results are basically consistent. Due to different disturbances on temperature field caused by different defects, the temperature differences on the top of solder balls are obvious .The thermal image of the solder (1), (2) and (3) at 0.2 s is shown in Figure 15a. Figure 15b is obtained through gray enhancement algorithm and Gauss low-pass filtering processing. It can be seen from the Figure 15b, on the top of the crack solder (1) and the intact solder (2), the temperature distributions are both uniform. However, the top colour of the intact solder is brighter than that of the crack solder which means the temperature is higher on the top of the intact solder. For the void solder, a circular distribution with the centre temperature being lower than the edge temperature is formed. This shows the effect of the crack on the eddy current field makes the top temperature field in low temperature condition and the effect of the void on the eddy current field makes the top temperature field in high temperature condition. The results are consistent with simulation analysis in Section 3.1.

## 5. Conclusions and Future Work

This paper studies the detectability effect of solder ball defects on the temperature field based on ECPT. 3D mathematical models of induction heat are established with the numerical analysis tool. Based on the simulation results, the theoretical analysis and experimental verification, the following conclusions can be drawn:

1. ECPT technology can effectively detect fine defects in the mini-size object. As shown in Figure 3a, when a micron-scale defect such as crack, void, exists in solder balls, the temperature differences of solder balls are significant. Thus, fine defects can be detected effectively by ECPT.
2. ECPT technology can distinguish different defects of solder balls: crack, void or missing. As depicted in Figure 4, under the infrared camera, the annular phenomena with the dark middle region and bright edge region will appear due to the presence of void. However, the crack will result in dark spots on the solder top. If there are missing balls, the temperature of the defect area should be consistent with the temperature of the surrounding area because of no induction heat.
3. Both experimental results and the simulation results show that tiny flaws on micro structure is detectable by using ECPT technology.

Future work will be focused on the impact of the relative position of the induction coil and the solder ball on the eddy current density by the FEM tool and experiment. Meanwhile, the relationship between the defect size and position and temperature filed will also be further discussed.
